# Pseudohypertriglyceridemia: A Novel Case with Important Clinical Implications

**DOI:** 10.1155/2020/4609317

**Published:** 2020-08-06

**Authors:** Ankur Rughani, Kenneth Blick, Hui Pang, Monica Marin, Jonathan Meyer, Jeanie B. Tryggestad

**Affiliations:** ^1^Department of Pediatrics, Section of Diabetes and Endocrinology, University of Oklahoma Health Sciences Center, Oklahoma City, Okla, USA; ^2^Department of Pathology, University of Oklahoma Health Sciences Center, Oklahoma City, Okla, USA; ^3^Department of Pediatrics, Section of Genetics, University of Oklahoma Health Sciences Center, Oklahoma City, Okla, USA

## Abstract

Pseudohypertriglyceridemia is an overestimation of serum triglyceride levels that may incorrectly lead to a diagnosis of hypertriglyceridemia. Glycerol kinase deficiency is a condition in which glycerol cannot be phosphorylated to glycerol-3-phosphate, resulting in elevated levels of serum glycerol. Laboratory assays that measure triglycerides indirectly may be affected by elevated glyerol levels and incorrectly report serum tryglyceride levels. We present a case of a novel missense mutation in the *GK* gene leading to isolated glycerol kinase deficiency and pseudohypertriglyceridemia in a male infant of a mother with gestational diabetes. This paper reviews glycerol kinase deficiency, describes the challenges in diagnosing pseudohypertriglyceridemia, and provides suggestions on improving diagnostic accuracy. Additionally, a potential maternal-fetal interaction between gestational diabetes and glycerol kinase deficiency is discussed.

## 1. Introduction

Pseudohypertriglyceridemia is an overestimation of serum triglyceride levels due to laboratory assays that measure free glycerol concentrations instead of triglycerides directly [1]. Specifically, laboratory assays commonly used today rely on microbial lipase enzymes to hydrolyze triglycerides into free fatty acids and glycerol, followed by enzymatic quantitation and therefore measure monoglycerides, diglycerides, triglycerides, and free glycerol [2]. Chemical methods used in the past to measure triglycerides directly by separating phospholipids from glycerol are too labor-intensive for automated commercial laboratories [3]. Consequently, conditions presenting with elevated levels of endogenous or exogenous free glycerol, such as glycerol kinase deficiency, result in an overestimation of serum triglycerides. While triglycerides are an important marker of metabolism, cardiovascular, and pancreatic health, overestimation of the triglyceride levels can lead to inappropriate and futile medical therapy which unnecessarily attempts to lower triglyceride levels in efforts to reduce the risk of cardiometabolic disease [4].

Glycerol kinase deficiency (GKD) is a rare X-linked recessive condition due to a mutation in the *GK* gene, which is found on the short (*p*) arm of chromosome X at position 21.2 [5]. The mutation leads to a condition in which glycerol cannot be phosphorylated to glycerol-3-phosphate and, therefore, cannot be used as a substrate in gluconeogenesis [6]. Glycerol blanking has occasionally been used to control the overestimation of triglycerides, but this method is not always available [7].

## 2. Case Presentation

The subject was a term male infant born to a previously healthy mother with gestational diabetes mellitus (GDM) requiring insulin (type A2). He was admitted to the NICU for hypoxic ischemic encephalopathy secondary to meconium aspiration. He was born at the 41^st^ week of gestation by emergency Caesarean section for non-reassuring fetal heart pattern after failed induction. APGAR scores after birth were 2 at 1 minute of life, 6 at 5 minutes of life, and 7 at 10 minutes of life. The infant was intubated at 10 minutes of life due to severe respiratory distress as a result of severe pulmonary hypertension and underwent therapeutic hypothermia for 3 days. On physical examination, the infant's birth weight was 5430 grams (WHO *Z*-score +3.6, large for gestational age), and he did not have any dysmorphic features, midline defects, or organomegaly. His neurologic exam was remarkable for mild hypotonia and several beat clonus at the ankles. The infant received total parenteral nutrition for 17 days, including intralipids for the first 7 days. Routine laboratory tests incidentally noted elevated triglycerides (961 mg/dL) on day of life 7, after which intralipids were discontinued. His lipid panel was otherwise unremarkable given his age: total cholesterol was 69 mg/dL, non-HDL cholesterol was 44 mg/dL, and HDL cholesterol was 28 mg/dL. The rest of his workup, including electrolytes, glucose, liver function, thyroid function, and essential fatty acids, were within normal limits for age. The newborn screens at 24 hours and 2 weeks of life were normal. Posthypothermia MRI of the brain was unremarkable. Ophthalmic exam was significant for bilateral foveal hemorrhages but negative for lipemia retinalis. No fat necrosis was identified. The infant received a gastrostomy tube due to poor oral intake. There was no known family history of dyslipidemia.

The infant was transitioned from parenteral to enteral nutrition with high medium-chain triglycerides (MCT) formula. He was initially on casein-based formula with 55% MCT and then placed on casein-based formula with 84% MCT. However, serum triglycerides remained relatively unchanged despite changes in formula, persisting between 900 mg/dL and 1280 mg/dL. Sequencing of genes associated with chylomicronemia (*APPL1, BLK, CEL, GCK, HNF1A, HNF1B, HNF4A, INS, KCNJ11, KLF11, NEUROD1, PAX4, PDX1, APOA5, APOC2, GPIHBP1, LMF1,* and *LPL)* showed no variants. A chromosomal microarray was also unremarkable with no regions of homozygosity. Whole exome sequencing revealed a missense variant c.763 G > A (p.Gly255Arg) in the *GK* gene (NM_000167.5) consistent with isolated GKD, resulting in pseudohypertriglyceridemia. This variant in exon 9 leads to the replacement of the amino acid glycine at position 255 with arginine in a region that is highly conserved across 13 species. In silico analysis tools predict this variant to be damaging to the glycerol kinase function or structure [8–10]. Segregation analysis was not performed per family preference. Corrected triglycerides, after blanking, were subsequently measured and found to be 82 mg/dL, and serum glycerol was 101 mg/dL (reference range 5–20 mg/dL [11]), confirming pseudohypertriglyceridemia. Glycerol kinase enzyme activity has not been assessed.

## 3. Discussion

The etiology of hypertriglyceridemia in children includes (i) familial hypertriglyceridemia, an autosomal dominant condition that results from hepatic secretion of large VLDL particles that are triglyceride-rich; (ii) familial chylomicronemia syndrome, which is caused by defective lipoprotein lipase activity; (iii) fat necrosis, which may occur in infants undergoing therapeutic hypothermia; and (iv) iatrogenic causes such as the administration of intralipids [12, 13]. When the history and biochemical findings are not consistent with any of these etiologies in a patient with elevated triglycerides, pseudohypertriglyceridemia secondary to glycerol kinase deficiency should be considered [1].

GKD, first described in 1978, is an X-linked recessive disorder due to a variant in the *GK* gene on Xp21 in which glycerol—a product of lipolysis—cannot be phosphorylated to glycerol-3-phosphate; therefore, it can neither be used as a substrate in gluconeogenesis nor be esterified to free fatty acids [14, 15]. Biochemically, this results in elevated serum and urine glycerol levels [14]. There are currently three accepted forms of GKD corresponding with their symptoms: complex, isolated symptomatic, and isolated benign [6, 14]. Complex GKD involves two additional genes that are contiguous with *GK*, namely, *DAX1* and *DMD.* The deletion of *DAX1* leads to primary adrenal insufficiency secondary to congenital adrenal hypoplasia manifested by signs of glucocorticoid and mineralocorticoid deficiencies such as hypoglycemia, hyperpigmentation, and electrolyte disturbances during adrenal crises and in periods of stress and illness; the involvement of the *DMD* gene, known to cause Duchenne muscular dystrophy, leads to progressive muscular disease [14, 16]. Additionally, patients with complex GKD present with triangular facies with an “hourglass” midface appearance, mental retardation, emesis, and metabolic acidosis [16].

In contrast to complex GKD, isolated GKD may be either symptomatic or asymptomatic; however, the phenotype may change over time, suggesting no strict genotype-phenotype correlation [17]. The symptomatic form, also referred to as the juvenile form, presents with intermittent emesis, ketosis and acidosis, lethargy, hypoglycemia, unconsciousness, and seizures in early childhood, but symptoms appear to improve with time [17]. The variation of symptoms between childhood and adulthood has been attributed to a relative glucose deficit in children in which hepatic glucose output is unable to meet the metabolic demands during periods of prolonged starvation or catabolism [17]. Management of isolated GKD, therefore, is focused on avoiding prolonged fasting and on treatment with dextrose-containing fluids in hospitalized patients who are unable to tolerate oral intake. Long term, elevated glycerol levels do not appear to have a clinically significant negative impact on cardiovascular health, though they may contribute to insulin resistance making monitoring for hyperglycemia and type 2 diabetes mellitus an important goal of surveillance [4].

This case has several implications. The missense mutation in the *GK* gene, c.763 G > A (p.Gly255Arg), leading to isolated glycerol kinase deficiency is a novel variant not previously reported to cause disease. Furthermore, our case illustrates the clinical challenges of diagnosing pseudohypertriglyceridemia. Glycerol blanking differentiates pseudohypertriglyceridemia from true hypertriglyceridemia but is not widely available. The consequences of unidentified pseudohypertriglyceridemia include overtreatment and pursuing additional, often expensive, specialized testing, including genetic sequencing, unnecessarily. Given the uncertain prevalence of isolated GKD and its asymptomatic nature, it is likely underdiagnosed and often incidentally discovered, leading to needless medical therapy, potentially unnecessary testing of family members, as well as increased patient anxiety. To avoid these problems, one should consider requesting the laboratory either blank for glycerol or otherwise correct for hyperglycerolemia in cases of hypertriglyceridemia without other lipid abnormalities or in asymptomatic cases. Alternatively, laboratories should consider reflexing to glycerol blanking in cases of hypertriglyceridemia in which the total cholesterol levels are unusually normal, e.g., by estimation using the Friedewald equation [18]. The addition of the *GK* gene to commercially available chylomicronemia or hypertriglyceridemia gene sequencing panels may also aid in making the appropriate diagnosis sooner rather than later. In this case, for instance, the diagnosis of GKD would have been made a lot sooner, and with less expense had the *GK* gene been included in the chylomicronemia gene panel. Instead, whole exome sequencing was pursued at added expense and resource utilization after the chylomicronemia panel was unrevealing.

It is noteworthy that the proband's mother was diagnosed with GDM requiring insulin. It has previously been noted that *GK* likely plays an important role in insulin signaling, insulin resistance, and type 2 diabetes mellitus [19]. Furthermore, a maternal-fetal association has previously been reported between GDM in a mother who was a GKD carrier and her male fetus with isolated GKD [20]. A similar maternal-fetal genotype interaction has been reported between maternal fatty liver disease and fetuses with 3-hydroxyacyl-CoA dehydrogenase deficiency [21]. Although the maternal genotype here is unknown, the intriguing finding of GDM in a young and previously healthy mother highlights the potential maternal-fetal interaction between GDM and GKD previously noted [20].

In conclusion, this case highlights the diagnostic challenges of pseudohypertriglyceridemia, presents a novel mutation that leads to isolated GKD, and discusses the expected course, management, and prognosis of GKD, while emphasizing the importance of glycerol blanking and the addition of *GK* gene to chylomicronemia or hypertriglyceridemia gene sequencing panels. Additionally, this case underscores the need for further investigation into the maternal-fetal interaction between GDM and GKD.

## Abbreviations

GKD: Glycerol kinase deficiency
WHO: World Health Organization
MCT: Medium-chain triglycerides
GDM: Gestational diabetes mellitus.
